# GLDC mitigated by miR-30e regulates cell proliferation and tumor immune infiltration in TNBC

**DOI:** 10.3389/fimmu.2022.1033367

**Published:** 2022-10-07

**Authors:** Huaying Xie, Tingting Yan, Xinxin Lu, Yueyao Du, Shuguang Xu, Yu Kong, Liangjie Yu, Jian Sun, Liheng Zhou, Jun Ma

**Affiliations:** ^1^ Department of Radiation Oncology, Renji Hospital, School of Medicine, Shanghai Jiao Tong University, Shanghai, China; ^2^ Department of Breast Surgery, School of Medicine, Renji Hospital, Shanghai Jiao Tong University, Shanghai, China; ^3^ Department of Oncology, Ganzhou Women and Children’s Health Care Hospital, Ganzhou, China; ^4^ Department of Breast Surgery, Obstetrics and Gynaecology Hospital, Fudan University, Shanghai, China; ^5^ Eye Institute, Eye & Ear, Nose, and Throat (ENT) Hospital, Shanghai Medical College, Fudan University, Shanghai, China

**Keywords:** TNBC, GLDC, proliferation, miR-30e, tumor immune

## Abstract

**Background:**

TNBC, whose clinical prognosis is poorer than other subgroups of breast cancer, is a malignant tumor characterized by lack of estrogen receptors, progesterone hormone receptors, and HER2 overexpression. Due to the lack of specific targeted drugs, it is crucial to identify critical factors involved in regulating the progression of TNBC.

**Methods:**

We analyzed the expression profiles of TNBC in TCGA and the prognoses values of GLDC. Correlations of GLDC and tumor immune infiltration were also identified. CCK8 and BrdU incorporation assays were utilized to determine cell proliferation. The mRNA and protein levels were examined by using Real-time PCR and Western blot analysis.

**Results:**

In the present study, we analyzed the mRNA expression profiles of TNBC in TCGA and found that GLDC, a key enzyme in glycine cleavage system, was significantly up-regulated in TNBC tissues and higher expression of GLDC was correlated with a worse prognosis in TNBC. Moreover, the expression of GLDC was negatively correlated with macrophage and monocyte and positively correlated with activated CD4 T cell and type 2 T helper cell in TNBC. Overexpression of GLDC facilitated the proliferation of TNBC cells, whereas GLDC knockdown had the opposite effects. Additionally, miR-30e acts as a functional upstream regulator of GLDC and the inhibitory effects of miR-30e on cell proliferation were mitigated by the reintroduction of GLDC.

**Conclusions:**

These results imply that miR-30e-depressed GLDC acts as a tumor suppressive pathway in TNBC and provides potential targets for the treatment of TNBC.

## Introduction

Breast cancer is the most common cancer in women and remains the second leading cause of cancer death among women worldwide (1). Triple-negative breast cancer (TNBC), defined by a lack of expression of both estrogen (ER) and progesterone receptor (PR) as well as human epidermal growth factor receptor 2 (Her2), is the most aggressive subgroup of breast cancer and accounts for 12–18% of all invasive breast cancers (2). Due to the lack of therapeutic targets, the clinical prognosis of TNBC is also worse than other subgroups of breast cancer. Therefore, it is of enormous therapeutic interest to explore the key molecules involved in affecting the development and diagnosis of TNBC and elucidate the regulatory mechanisms.

Glycine Decarboxylase (GLDC) is a key enzyme in glycine cleavage system, which can convert glycine into a carbon unit. Abnormal regulation of glycine decarboxylase is related to the occurrence of various human tumors, but roles of GLDC in different cancers are not always consistent (3). Liu et al. demonstrated that rapamycin complex 1 (mTORC1) signal inhibits GLDC acetylation by inducing the transcription of deacetylase SIRT3 (SIRT3) and GLDC acetylation inhibits glycine catabolism, pyrimidine synthesis and glioma (4). A report has shown that the expression of GLDC is significantly increased in MYCN amplified neuroblastoma tumors and cell lines, and GLDC plays a key role in maintaining the proliferation of neuroblastoma cells (5). In lung cancer, it has been reported that GLDC induces significant changes in glycolysis and glycine/serine metabolism, leading to changes in pyrimidine metabolism, thereby regulating the proliferation of non-small cell lung cancer cells. Clinically, abnormal activation of GLDC is associated with poor survival in patients with lung cancer (6). However, other studies have shown that GLDC inhibits the metastasis and is positively correlated with the overall survival by acting as a tumor suppressor in HCC (7, 8). Until now, roles of GLDC in TNBC are not clear and need to be determined.

In this research, we examined the regulatory roles and clinicopathologic significance of GLDC in TNBC and determined the underlying mechanism. Our results showed that GLDC was significantly increased in TNBC tissues and higher expression of GLDC was correlated with a worse prognosis. The expression of GLDC was closely correlated with several types of immune cells and GLDC facilitated the proliferation of TNBC cells. Moreover, miR-30e negatively regulated the expression of GLDC by acting as a functional upstream regulator. The findings elucidate an important regulatory mechanism and might provide potential therapy targets for TNBC.

## Material and methods

### Materials

MDA-MB-231 was purchased from the American Type Culture Collection (ATCC). MiR-30e mimics and the miR-30e inhibitor (anti-miR-30e) were synthesized in Ribobio (Guangzhou, China). The primary antibody for Rabbit anti-GLDC was bought from Abcam (ab97625, 1:500). Goat anti-Rabbit IgG was got from Cell Signaling Technology (7074, 1:5000). Dulbecco’s modified Eagle’s medium (DMEM) and fetal bovine serum (FBS) were purchased from hyclone and Gibco (Thermo Fisher Scientific), respectively. Bromodeoxyuridine (BrdU) incorporation assay kit was got from Roche Diagnostics (IN, USA).

### Cell viability assay

Cell viability was examined by using the cell counting kit-8 (CCK-8). Briefly, 3000 cells/well were cultured in 96-well plate at 37°C for 24 hours. Then 10 μl of CCK-8 was added to each well in the plate. After 2 hours, we utilized a microplate reader (Thermo Scientific, Rockford, IL, USA) to determine the absorbance at 490nm.

### Bromodeoxyuridine incorporation assay

Cell proliferation was determined by using a 5-bromo-2’-deoxyuridine (BrdU) kit to detect the BrdU incorporation. We first cultured the cells into a 96-well plate at a density of 5000 cells/well. After 12 hours, 10 μl BrdU labeling solution was added into each well and we incubated the plates at 37°C for 24 hours. Then 200 μl anti-BrdU peroxidase solutions were added to label cells for 1.5 hours at room temperature. Finally, we washed the sample with washing solution and then added 100 μl tetramethylbenzidine substrate solutions to each well at room temperature for 30 minutes. A microplate reader (Thermo Scientific) was utilized to determine the absorbance at 450nm.

### Real-time PCR and Luciferase reporter assay

Real-time PCR and Luciferase reporter assay were performed as our previous descriptions (9). Primers for GLDC and β-actin were designed and listed as follows: GLDC forward, 5’−CTGCTGTGCTACTGACCTTTT−3’ and reverse, 5’−CCAGGCATCATTCTCACCAAG−3’; β-actin forward, 5’−CATGTACGTTGCTATCCAGGC−3’ and reverse, 5’−CTCCTTAATGTCACGCACGAT−3’. Specific primers and Taqman probes for microRNA analysis were purchased from Applied Biosystems. The mRNA levels of beta-Actin and snRNA U6 were used as the internal normalization control, respectively. The kit for Dual-Luciferase Reporter Assay System was obtained from Promega Corporation and the experiment was performed as the protocol provided by the supplier.

### Western blot analysis

We performed western blot to determine the protein levels of target proteins. Briefly, the cell lysates were quantified with the BCA methods. The protein extracts (50 μg) were separated by SDS PAGE electrophoresis and then transferred to PVDF membranes. The membranes were blocked by 5% non-fat milk and then incubated with primary antibodies at 4°C for 12 hours. After washing five times with PBS-T for 30 min, the membranes were incubated with secondary antibodies for 1 hours at room temperature. PBS-T was used to wash the membranes again for five times. Then the membranes were incubated with the ECL luminescence solution (Thermo Scientific) and the immunoreactive bands were acquired. Image J software was utilized to determine the optical density of the bands.

### Statistical analysis

Statistical analysis was performed by using GraphPad Prism v.9.0. The data for statistical analyses was obtained from at least three independent experiments and presented by mean ± standard error of the mean (SEM). Student’s t-test or one-way ANOVA followed by Dunnett’s test was appropriately applied for identifying the statistical significance. The Kaplan-Meier method was used to analyze the cumulative survival rate. The *P*-value was 0.05 or less was regarded as the statistically significant difference.

## Results

### Identification of differentially expressed genes in TNBC tissues and functional analysis

**Figure 1:**
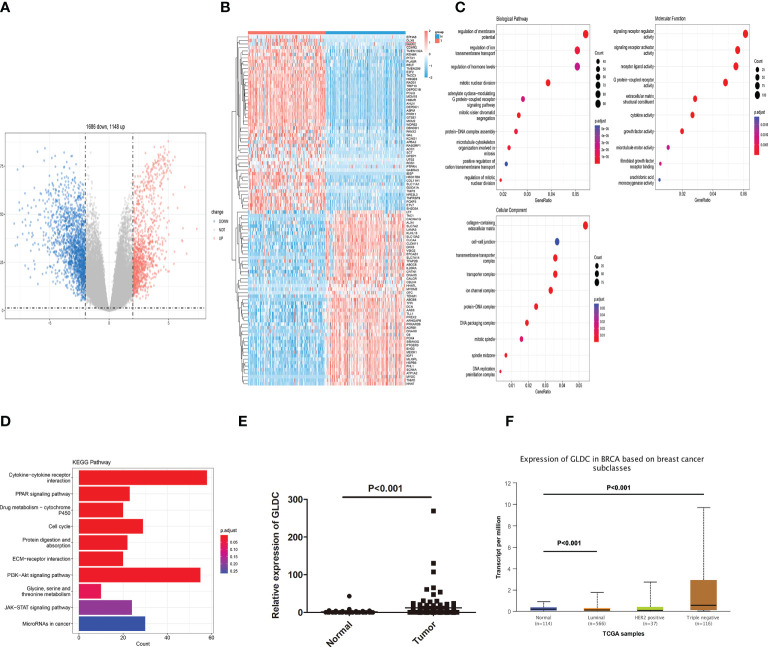
Identification of differentially expressed genes in TNBC tissues. **(A)** The overall distribution of differentially expressed genes by volcano plots. **(B)** The heat map of 100 differential genes. **(C)**: The biological processes, molecular function, and cellular component enriched by GO analysis. **(D)** The enriched KEGG pathways. **(E, F)** The expression of GLDC was significantly up-regulated in TNBC tissues compared with normal breast tissues by analyzing TCGA **(E)** and the UALCAN database **(F)**.

We first analyzed the mRNA expression profiles of TNBC in TCGA to determine the critical factors involved in affecting the progression of TNBC. As shown in **Figure 1A**, volcano plots were used to assess the gene expression variation and the overall distribution of differentially expressed genes (1148 upregulated genes and 1686 downregulated genes) between the TNBC tissues and normal breast tissue. 100 differential genes (containing 50 upregulated genes and 50 downregulated genes) are selected to draw the heat map (**Figure 1B**). Moreover, GO analysis and KEGG pathway analysis were performed by using all differentially expressed mRNAs. We found the biological processes (BP) enriched by GO analysis were regulation of ion transmembrane transport, hormone levels, mitotic relevant events. Molecular function (MF) of GO terms was enriched in signaling receptor regulator and activator activities, receptor ligand activity, G protein−coupled receptor activity, and growth factor activity. Cellular component (CC) of GO terms was enriched in collagen−containing extracellular matrix, cell−cell junction, and transmembrane transporter complex (**Figure 1C**). As shown in **Figure 1D**, the enriched KEGG pathways were shown. The results showed that some pathways directly related to affect the progression of cancer, such as cell cycle, PI3K-Akt signaling pathway, cytokine-cytokine receptor interaction and PPAR signaling pathway, were enriched. Moreover, we found that Glycine, serine, and threonine metabolism was enriched by KEGG pathway analysis. GLDC, which acts as a key enzyme in glycine cleavage system, was obviously up-regulated in TNBC tissues (**Figure 1E**). The consistent result that the expression of GLDC was increased in breast cancer, especially in TNBC tissues, was obtained by utilizing the UALCAN database (**Figure 1F**). These results indicate that GLDC might participate in regulating the progression and development of TNBC.

### The expression profiles of GLDC and its prognoses values in TNBC tissues

**Figure 2:**
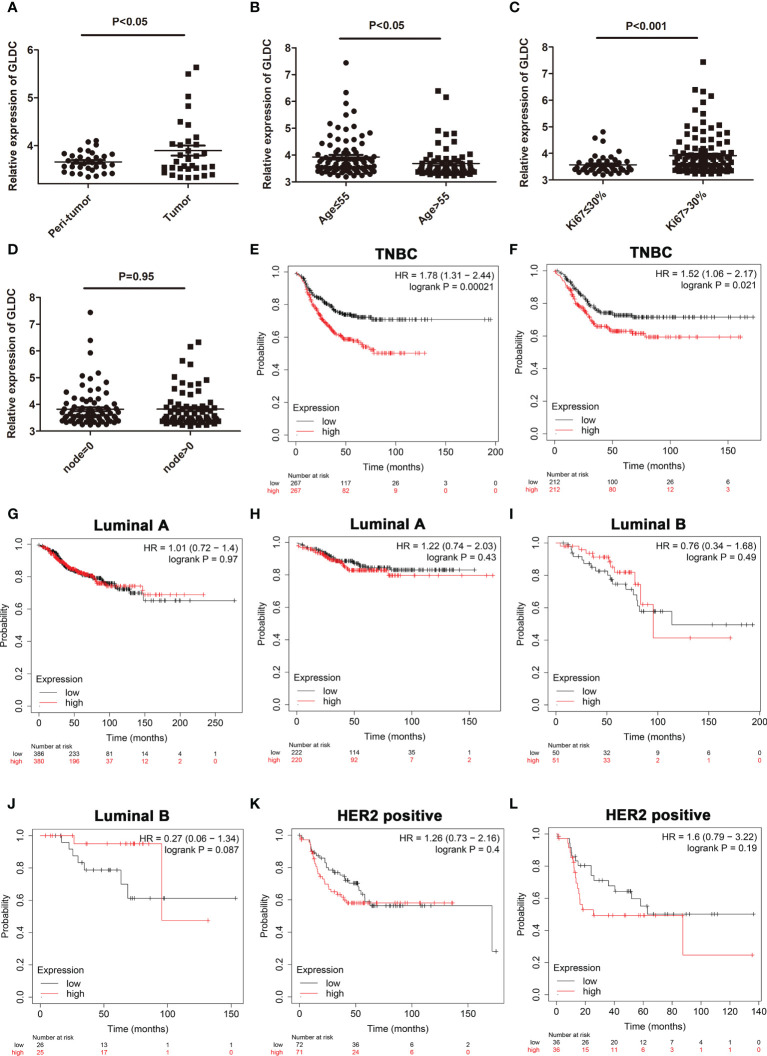
The expression profiles of GLDC and its prognoses value in TNBC tissues. **(A)** The expression of GLDC was significantly higher in the TNBC tissues compared with their adjacent normal tissues (n=33). **(B)** The expression of GLDC is higher in the patients younger than 55 years (age ≤ 55) than the patients older than 55 years (age>55). **(C)** GLDC expression in the patients with ki67 ≤ 30% was less than that with ki67>30%. **(D)** No significant differences were detected on the expression of GLDC between positive lymph nodes and no positive lymph nodes. **(E, F)** TNBC patients with high levels of GLDC were associated with the shorter RFS **(E)** and the worse DMFS **(F)**. **(G, H)** There were no significant differences on the RFS **(G)** and DMFS **(H)** between the low levels of GLDC and the high levels of GLDC in Luminal A breast cancer. **(I, J)** No significant differences were detected on the RFS **(I)** and DMFS **(J)** between the two groups in Luminal B breast cancer. **(K, L)** There were no significant differences on the RFS **(K)** and DMFS **(L)** between the two groups in HER2 positive breast cancer.

To confirm changes of GLDC in TNBC tissue, we also analyzed its expression from a GEO dataset (GSE76250). The results showed that the expression of GLDC was significantly higher in the TNBC tissues compared with their paired adjacent normal tissues (**Figure 2A**). We further examined the expression levels of GLDC in the TNBC tissues based on the clinicopathological variables. We found that the expression of GLDC is higher in the patients younger than 55 years (age ≤ 55) than the patients older than 55 years (age>55) (**Figure 2B**). GLDC expression in patients with ki67 ≤ 30% was lower than that with ki67>30% (**Figure 2C**). However, there are no significant differences on the expression of GLDC between positive lymph nodes and no positive lymph nodes (**Figure 2D**). Furthermore, the prognoses values of GLDC in TNBC were also examined by utilizing the Kaplan-Meier Plotter (10). We found that patients with the high levels of GLDC were associated with the shorter recurrence free survival (RFS) (median RFS time (months), 22.37 (high levels of GLDC) and 43 (low levels of GLDC) months, respectively; P < 0.001) and the worse distant metastasis free survival (DMFS) (median DMFS time (months), 26.63 (high levels of GLDC) and 38.6 (low levels of GLDC) months, respectively; P < 0.05) than the patients with low GLDC expression in TNBC (**Figures 2E, F** **)**. In addition, we also examined the prognoses values of GLDC in other types of breast cancer. We found that there were no significant differences on the RFS and DMFS between the low levels of GLDC and the high levels of GLDC in Luminal A, Luminal B and HER2 positive types of breast cancer (**Figures 2G–L**). The results imply that GLDC might be considered as a potential predictive molecule of prognosis for TNBC.

### The relationship between GLDC and tumor immune infiltration

**Figure 3:**
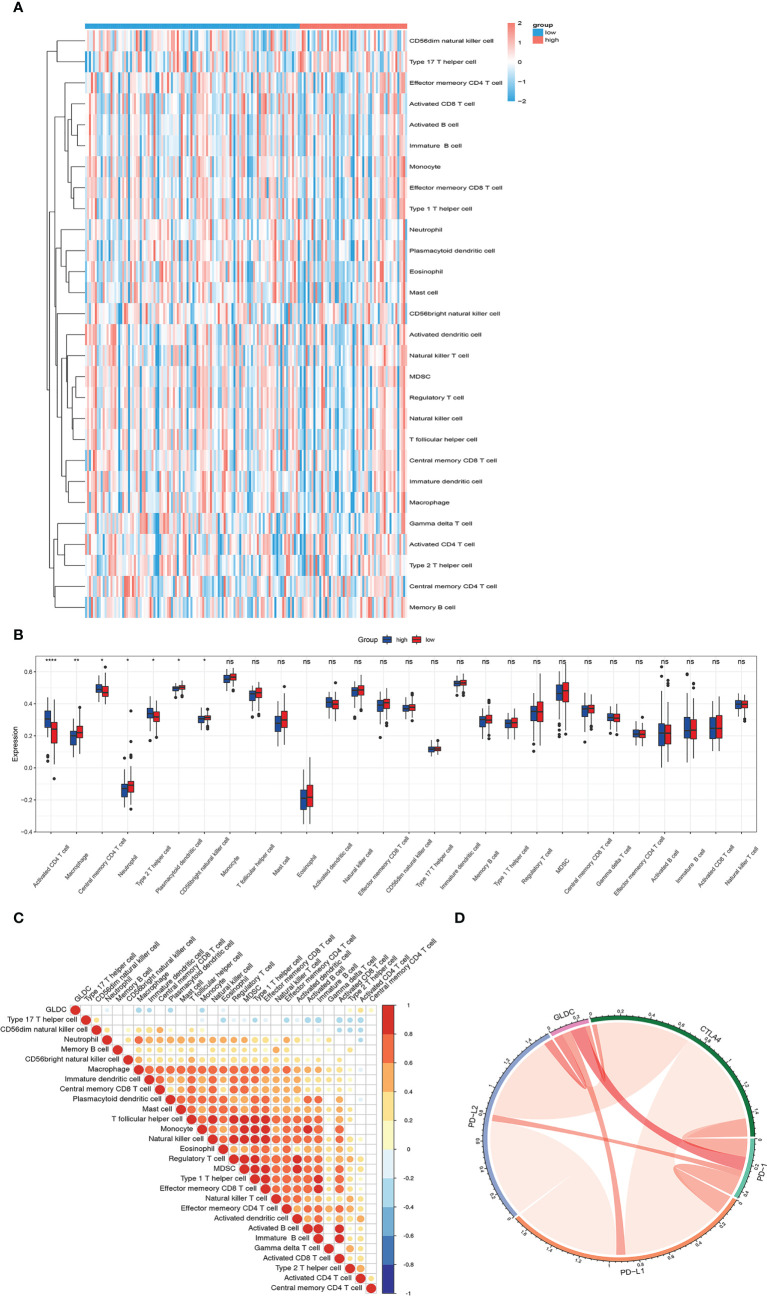
The relationship between GLDC and tumor immune infiltration. **(A)** The infiltration abundance of immune cells based on the levels of GLDC in TNBC patients. **(B)** High levels of macrophage, neutrophil, CD56 bright natural killer cell and plasmacytoid dendritic cell and low levels of activated CD4 T cell, central memory CD4 T cell, and type 2 T helper cell were acquired in the group with low expression of GLDC in TNBC. **(C)** The expression of GLDC was negatively correlated with macrophage, plasmacytoid dendritic cell, and monocyte and positively correlated with activated CD4 T cell and type 2 T helper cell. **(D)** No significant correlations were observed between GLDC and the four immune checkpoint molecules in TNBC. *P < 0.05, **P < 0.01, ***P < 0.001, and ns indicates P>0.05.

Previous studies have shown that tumor immune microenvironment plays an important role in affecting tumor growth. The immune cells and immune-related signaling pathways are involved in regulating the progression of cancer and the response to cancer therapy (11, 12). Therefore, we future examined whether GLDC was correlated with the tumor immune infiltration in TNBC. As shown in **Figures 3A, B**, we analyzed the infiltration abundance of immune cells in TNBC patients based on the levels of GLDC. The results showed that activated CD4 T cell, central memory CD4 T cell, and type 2 T helper cell were significantly enriched in the group with high expression of GLDC in TNBC, whereas high levels of macrophage, neutrophil, CD56 bright natural killer cell and plasmacytoid dendritic cell were acquired in the TNBC patients with low GLDC expression. Moreover, we further examined the relationship between GLDC expression and immune cell types. We found that the expression of GLDC was negatively correlated with macrophage, plasmacytoid dendritic cell, and monocyte, while GLDC expression was positively correlated with activated CD4 T cell and type 2 T helper cell (**Figure 3C**). By analyzing the relationship between GLDC and four immune checkpoint molecules (CTLA4, PD-1, PD-L1, and PD-L2), no significant correlations were acquired between GLDC and the four immune checkpoint molecules in TNBC (**Figure 3D**). These results imply that GLDC likely has the regulatory effects on tumor immune microenvironment in TNBC.

### GLDC facilitates cell proliferation in TNBC cells

**Figure 4:**
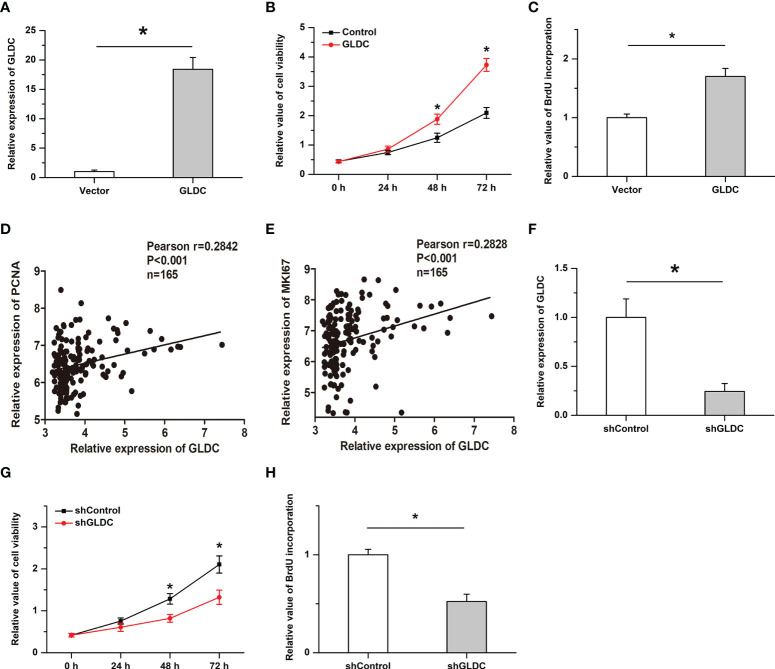
Cell proliferation is promoted by GLDC in TNBC cells. **(A)** Real-time PCR was utilized to determine the overexpression efficiency. **(B, C)** Overexpression of GLDC increased cell viability **(B)** and facilitated BrdU incorporation **(C)**. **(D, E)** GLDC expression was positively correlated with the expression of PCNA **(D)** and MKI67 **(E)** in TNBC tissues. **(F)** Knockdown efficiency was determined by Real-time PCR. **(G, H)** Knockdown of GLDC mitigated cell viability **(G)** and repressed BrdU incorporation **(H)**. *P < 0.05.

We then examined the effects of GLDC on the growth of TNBC cells. Overexpression of GLDC were established in MDA-MB-231 (a cell line of TNBC). As shown in **Figure 4A**, the overexpression efficiency was verified by real-time PCR. We found that cell viability was significantly increased by GLDC overexpression (**Figure 4B**). Overexpression of GLDC facilitated BrdU incorporation into newly synthesized DNA (**Figure 4C**). Consistently, the expression of GLDC was positively correlated with the expression of PCNA and MKI67 (two markers of cell proliferation) in TNBC tissues (**Figures 4D, E** **)**. Furthermore, we also knocked down the expression of GLDC in TNBC cells to confirm its physiological function (**Figure 4F**). The results showed that the cell viability was mitigated by the knockdown of GLDC (**Figure 4G**). GLDC knockdown significantly repressed BrdU incorporation (**Figure 4H**). The results indicate that GLDC positively regulated the proliferation of TNBC cells.

### GLDC is negatively regulated by miR-30e in TNBC

**Figure 5:**
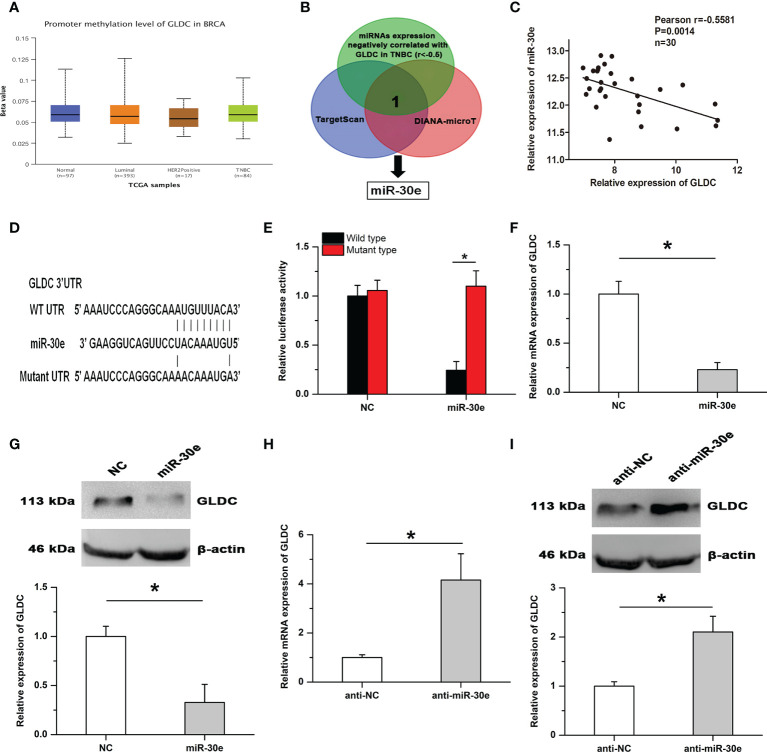
GLDC is negatively regulated by miR-30e in TNBC. **(A)** There was no significant difference on the promoter methylation level of GLDC between TNBC and normal breast tissues. **(B)** The schematic diagram of the protocol utilized for identifying the potential functional regulator of GLDC. **(C)** The expression of GLDC was negatively correlated with miR-30e expression in the same TNBC tissues. **(D)** The potential binding site of miR-30e conserved in the 3′UTR of GLDC. **(E)** Treatment with miR-30e led to the decreased luciferase activity of the WT (wild type) group. **(F, G)** MiR-30e significantly depressed the mRNA **(F)** and protein **(G)** expression of GLDC in TNBC cells. **(H, I)** Treatment with anti-miR-30e led to the increased expression of GLDC at both mRNA **(H)** and protein **(I)** levels. *P < 0.05.

By using the UALCAN database, we found that there was no significant difference on the promoter methylation level of GLDC between TNBC and normal breast tissues (**Figure 5A**). It is widely accepted that microRNAs play important roles in affecting the expression of target genes. To determine the mechanism of GLDC up-regulation in TNBC, we examined whether microRNAs might participate in regulating the expression of GLDC in TNBC. The result showed that miR-30e, a potential binding microRNA of GLDC, was negatively correlated with the expression of GLDC in the same TNBC tissues (GSE59595) (r=-0.5581, P=0.0014) (**Figures 5B, C** **)**. As shown in **Figure 5D**, the mutated UTR, which was utilized in the luciferase reporter assay, was constructed based on the potential binding site of miR-30e conserved in the 3′UTR of GLDC. Results of luciferase reporter assay showed that the luciferase activity of the WT (wild type) group was significantly reduced by the treatment with miR-30e, whereas there was no detectable change on the luciferase activity of the MT (mutant type) group (**Figure 5E**). Moreover, miR-30e obviously mitigated the mRNA and protein expression of GLDC in TNBC cells (**Figures 5F, G** **)**. Treatment with anti-miR-30e led to the increased expression of GLDC at both mRNA and protein levels (**Figures 5H, I** **)**. These results indicate that miR-30e acts as a functional upstream regulator of GLDC in TNBC.

### The inhibitory effects of miR-30e on cell proliferation are attenuated by GLDC

**Figure 6:**
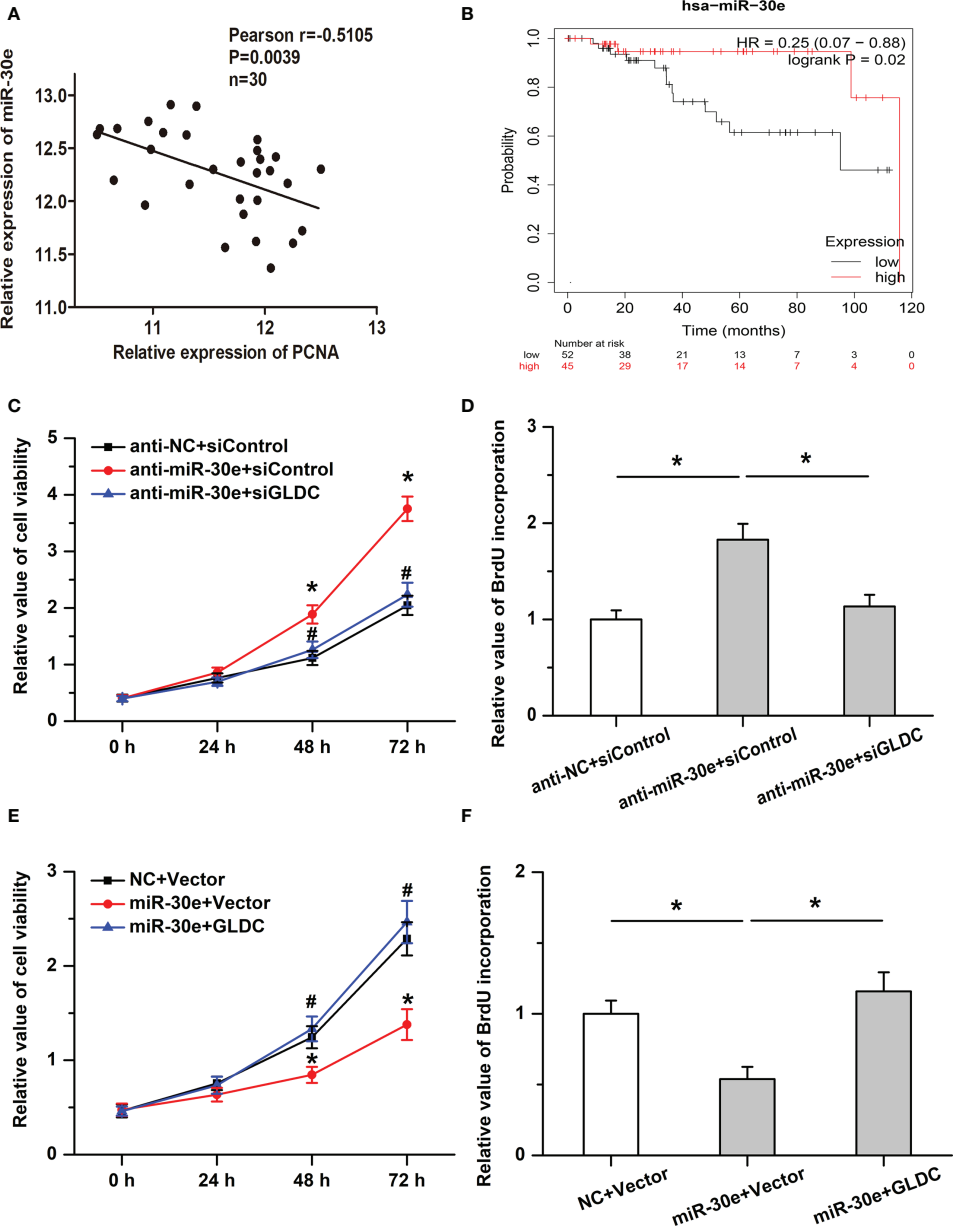
The inhibitory effects of miR-30e on cell proliferation are mitigated by GLDC. **(A)** The expression of miR-30e was negatively correlated with PCNA expression in the same TNBC tissues. **(B)** High levels of miR-30e were associated with better overall survival. **(C, D)** Treatment with anti-miR-30e increased cell viability **(C)** * indicates p < 0.05 compared with anti-NC+siControl group, # indicates p < 0.05 compared with anti-miR-30e+siControl group and promoted BrdU incorporation. **(D)**, which was attenuated by the knockdown of GLDC. **(E, F)**: Restoration of GLDC depressed the regulatory effects of miR-30e on cell viability **(E)** * indicates p < 0.05 compared with NC+Vector group, # indicates p < 0.05 compared with miR-30e+Vector group and BrdU incorporation **(F)**.

We then examined the roles of miR-30e in cell proliferation in TNBC. Our results showed that the expression of miR-30e was negatively correlated with PCNA expression in the same TNBC tissues (**Figure 6A**) and high levels of miR-30e were associated with better overall survival (OS) (median OS time (months), 115.73 (high levels of miR-30e) and 95.13 (low levels of miR-30e) months, respectively; P = 0.02 (**Figure 6B**). Treatment with anti-miR-30e increased cell viability and promoted BrdU incorporation, which was attenuated by the knockdown of GLDC (**Figures 6C, D** **)**. Moreover, the inhibitory effects of miR-30e on cell proliferation were attenuated by the restoration of GLDC in TNBC cells (**Figures 6E, F** **)**. These results imply that miR-30e inhibits the proliferation of TNBC cells, at least in part, by targeting GLDC.

## Discussion

TNBC, as the most aggressive subgroup of breast cancer, is lack of specific targeted drugs and the prognosis is far less than expected. Until now, pathological processes of TNBC remain largely unknown and still need to be determined. In the present study, our results showed that GLDC, which was significantly up-regulated in cancer tissues, facilitated cell proliferation and was negatively correlated with the RFS and DMFS in TNBC. The expression of GLDC was negatively correlated with macrophage and monocyte and was positively correlated with activated CD4 T cell and Type 2 T helper cell. Moreover, GLDC serves as a downstream target of miR-30e in TNBC and attenuated the inhibitory effects of miR-30e on cell proliferation. The results imply an important underlying mechanism of GLDC-regulated cell proliferation and tumor immune infiltration in TNBC.

An important finding of this research is that GLDC promotes cell proliferation and could be considered as a potential predicting factor for prognosis in TNBC. Although GLDC has been demonstrated to participate in regulating the development of tumors in some types of cancers, its roles in different cancers are controversial and not always consistent. Previous studies have shown that GLDC overexpression or its gene alternative splicing enhances cellular transformation and tumorigenesis and is correlated with poorer survival in non-small cell lung cancer (NSCLC) (6, 13). Inhibition of GLDC transcript represses cell proliferation and colony formation in NSCLC and prostate cancer cells (14, 15). GLDC knockdown mitigates cell proliferation and tumorigenicity *via* causing G1 arrest in MYCN-amplified neuroblastoma cells (5). Knockdown of glycine decarboxylase represses the growth of the tumor by regulating mitochondrial protein lipoylation in hepatocellular carcinoma (HCC) (16). On the contrary, it is reported that GLDC negatively regulates the migration and invasion of HCC cells *in vivo* and *in vitro* (7). The overall survival is better in the group with high expression of GLDC in HCC and overexpression of GLDC obviously facilitates cell autophagy and depresses intrahepatic metastasis (8). However, roles of GLDC in TNBC are still unknown. In the present study, our results showed that GLDC, significantly upregulated in cancer tissues, was correlated with a worse prognosis related to RFS and DMFS in TNBC. Overexpression of GLDC promoted cell proliferation, whereas GLDC knockdown had the opposite effects. Furthermore, it is widely accepted that tumor immune microenvironment is involved in affecting the development of cancers and the response to cancer therapy (12). Our results showed that several immune cells were significantly enriched or decreased in the TNBC patients with high levels of GLDC. The expression of GLDC was negatively correlated with macrophage and monocyte, while GLDC expression was positively correlated with activated CD4 T cell and type 2 T helper cell in TNBC. These results imply that GLDC likely serves as an oncogenetic factor in the progression of TNBC by regulating cell proliferation and tumor immune microenvironment.

Another important finding of this research is that miR-30e acts as a functional upstream regulator of GLDC in TNBC. It is known to all that dysregulation of microRNAs is a critical cause in the initiation and progression of various diseases. MicroRNAs are involved in regulating several cellular physiological functions of cancer cells, such as cell proliferation, survival and metastasis, by affecting the expression of target genes. MiR-30e, a multifunctional microRNA, has been reported to be involved in regulating the development of tumors. Previous studies have shown that miR-30e acts as a tumor suppressor and inhibits cell proliferation and metastasis in some cancers, including hepatocellular carcinoma (17), squamous cell carcinoma of the head and neck (18), colorectal cancer (19). However, other studies have reported that miR-30e promotes the progression and malignant processes of cancers, for instance, esophageal cancer (20), lung adenocarcinoma (21). Roles of miR-30e in breast cancer are also controversial. MiR-30e represses tumor growth, bone metastasis and chemosensitivity to paclitaxel in breast cancer (22, 23). Conversely, Overexpression of miR-30e-decreased expression of Tumor Suppressor Candidate 3 (TUSC3) leads to increased proliferation and migration of breast cancer cells (24). To date, the precise effects of miR-30e on TNBC are still inconclusive. Our results showed that miR-30e was positively associated with overall survival and negatively regulated cell proliferation in TNBC. The inhibitory effects of miR-30e on cell proliferation were attenuated by the restoration of GLDC. The results indicate that miR-30e-repressed GLDC defines a potentially suppressive pathway in TNBC. Although we have demonstrated that GLDC mitigated by miR-30e regulates cell proliferation and tumor immune infiltration in TNBC, the regulatory mechanisms remain unknown and will be determined in future studies. Additionally, we will further validate the regulatory effects of GLDC in TNBC and explore the possibility of GLDC as a potential therapeutic target for TNBC by utilizing more clinical samples and *in vivo* studies.

## Conclusion

In summary, this research implies that GLDC, increased in the TNBC tissues, facilitates cell proliferation and is correlated with the poor prognosis in TNBC as an oncogenetic factor. Moreover, miR-30e acts as a functional upstream regulator of GLDC in TNBC. The findings demonstrate the important regulatory effects of GLDC in TNBC, which might provide potential targets for improving the molecular therapy of TNBC.

## Data availability statement

The original contributions presented in the study are included in the article/supplementary material. Further inquiries can be directed to the corresponding authors.

## Author contributions

JM, LZ, and JS designed this study. HX, XL, YK and LY analyzed the data. HX, TY and JM wrote the manuscript and performed the experiments. All authors contributed to the article and approved the submitted version.

## Funding

This study was supported by the National Natural Science Foundation of China (82072923 and 82002777), Multidisciplinary Cross Research Foundation of Shanghai Jiao Tong University (YG2019QNA26).

## Conflict of interest

The authors declare that the research was conducted in the absence of any commercial or financial relationships that could be construed as a potential conflict of interest.

The reviewer GL declared a shared parent affiliation with the authors HX, TY, YD, SX, YK, LY, LZ to the handling editor at the time of the review.

## Publisher’s note

All claims expressed in this article are solely those of the authors and do not necessarily represent those of their affiliated organizations, or those of the publisher, the editors and the reviewers. Any product that may be evaluated in this article, or claim that may be made by its manufacturer, is not guaranteed or endorsed by the publisher.
